# Effect of Neonicotinoid Pesticides on Japanese Water Systems: Review with Focus on Reproductive Toxicity

**DOI:** 10.3390/ijms231911567

**Published:** 2022-09-30

**Authors:** Hayato Terayama, Kou Sakabe, Daisuke Kiyoshima, Ning Qu, Tsutomu Sato, Kaori Suyama, Shogo Hayashi, Kenichi Sakurai, Emiko Todaka, Chisato Mori

**Affiliations:** 1 Department of Anatomy, Division of Basic Medical Science, Tokai University School of Medicine, 143 Shimokasuya, Isehara, Kanagawa 259-1193, Japan; 2 Department of Environmental Preventive Medicine (Yamada Bee Company, Inc.), Center for Preventive Medical Sciences, Chiba University, 1-33 Yayoicho, Inageku, Chiba 263-8522, Japan; 3 Division of Environmental Preventive Medical Sciences, Center for Preventive Medical Sciences, Chiba University, 1-33 Yayoicho, Inageku, Chiba 263-8522, Japan; 4 Department of Bioenvironmental Medicine, Graduate School of Medicine, Chiba University, 1-8-1 Inohana, Chuo-ku, Chiba 260-8670, Japan

**Keywords:** neonicotinoid pesticide, river water, testis, ovary, toxicity, ecosystem

## Abstract

Neonicotinoid pesticides (NPs) are neurotoxic substances. They are highly effective as insecticides owing to their water solubility, permeability, and long-lasting activity. These molecules are structurally similar to nicotine and act as nicotinic acetylcholine receptor agonists. The administration of NPs to experimental animals reportedly causes neuromuscular and reproductive disorders. Moreover, recently reported problems caused by NPs include damage to land-dwelling creatures (such as mammals and birds), hydrobiology, and ecosystems. This review summarizes the recent reports on NP concentrations detected in river systems in several Japanese regions. These values were lower than the environmental standard values; however, seasonal variations were observed. Furthermore, reports on NP-induced testicular and ovarian toxicity were examined, revealing that the mechanism of injury is mainly driven by oxidative stress. The use of NPs is declining worldwide, except in Japan; therefore, continuous monitoring remains necessary.

## 1. Introduction

**Figure 1:**
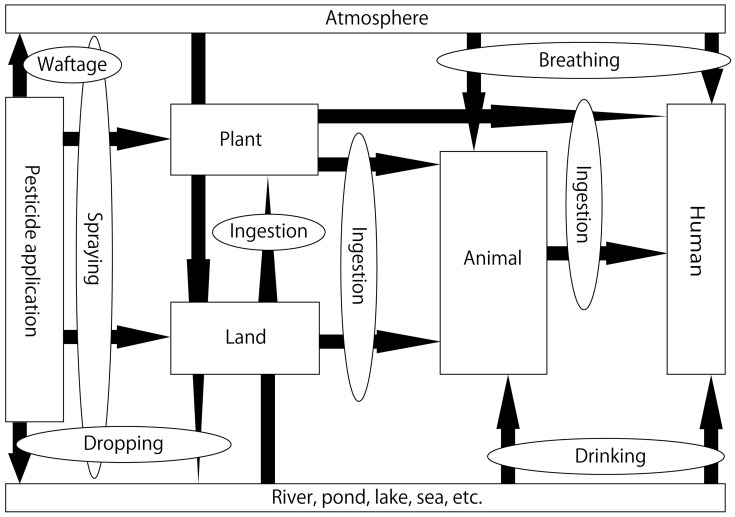
Circulation of pesticides in the ecosystem.

Agricultural chemicals have made great contributions toward ensuring the stable supply of agricultural products and reducing labor input. However, the problems of toxicity to biological life other than the target pests and environmental pollution are critical [1]. Agricultural chemicals are generally partially decomposed by soil organisms and light, while undecomposed pesticides flow out into water systems, such as rivers and seas, along with the flow of water in the soil and eventually reach the atmosphere. Pesticides also reach the ground with rainfall and flow into water systems [1,2]. Therefore, agricultural chemicals that are sprayed eventually end up in water systems [2] (Figure 1). Dichlorodiphenyltrichloroethane (DDT), an organochlorine pesticide, was used extensively in previous years. This led to its accumulation in insects; when these insects were consumed by birds and other animals, problems due to the bioaccumulation of this pesticide were reported [3,4]. As a result, environmental standard values were set to values lower than those in the past. Neonicotinoid pesticides (NPs) were introduced to the market in the 1990s and are among the most widely used pesticides today. Similar to conventional pesticides, NPs exert adverse effects on ecosystems and may bioaccumulate through the food chain [5,6,7,8]. The effects of NPs on humans and animals were compared, and the symptoms in humans and the observational results from animal experiments were similar. Additionally, effects on the central nervous, circulatory, body temperature, digestive, respiratory, and secretory systems were observed simultaneously. Few reviews have examined how neonicotinoid pesticides reach and affect mammals. The use of pesticides is of utmost importance for growing crops. Understanding the circulation of pesticides can help decide on the areas that require attention. This review summarizes reports documenting the concentrations of NPs in Japanese rivers and further outlines the toxic effects resulting from the ingestion of these pesticides, with a special focus on reproductive toxicity.

## 2. Literature Review

### 2.1. NPs

The seven commonly used NPs in Japan are imidacloprid, acetamiprid, nitenpyram, clothianidin, thiamethoxam, thiacloprid, and dinotefuran. NPs have a structure and action similar to that of nicotine; the structure comprises chlorine-containing chloronicotinyl insecticide and chlorine-free dinotefuran [9,10,11]. NPs are neurotoxic and have a high permeability, water solubility, and residual effects. These are absorbed from the surface of seeds, roots, leaves, and fruits and penetrate the plant body, thereby exerting their effects for an extended period. These are therefore extremely effective even as insecticides [12]. NPs are used for agricultural purposes, home gardening, and building pine lawns. These are also utilized widely for termite extermination, anti-termite treatment of building materials, veterinary drugs, and extermination of nuisance pests [13]. Fipronil, an insecticide belonging to the phenylpyrazole chemical group, has excellent permeability. Therefore, it is often studied in conjunction with the seven permeable NPs. Hence, this review also treats fipronil as an NP. NPs are neurotoxins that disrupt neurotransmission in target pests. They act as agonists of the nicotinic acetylcholine receptor (nAchR) and cause death by continuous nerve excitation [14]. In general, NPs are considered safe for the environment and are widely used.

The absorption rate of orally administered NPs in rats is ≥90%; it is mainly absorbed in the small intestine [13]. NPs that reach the blood are distributed throughout the body and cross the blood–brain barrier and placenta [13,14,15]. Among the NPs, acetamiprid exhibits the highest half-life in the brain, liver, and plasma at >240 min [13]. Among NPs, fipronil (male SD rat, 92 mg/kg; female SD rat, 103 mg/kg; male ICR mouse, 49 mg/kg; female ICR mouse, 57 mg/kg) has the lowest median lethal dose (LD_50_) concentration [16] followed by acetamiprid (male SD rat, 195 mg/kg; female SD rat, 146 mg/kg; male ICR mouse, 198 mg/kg; female ICR mouse, 184 mg/kg) and imidacloprid (male SD rats, 440 mg/kg; female SD rats, 410 mg/kg; male ICR mice, 100 mg/kg; female ICR mice, 98 mg/kg) [17,18]. In mammals, NPs are mainly metabolized by cytochrome P (CYP) enzymes in the liver microsomes and by aldehyde oxidase in the cytoplasm; approximately 95% of all NPs and their metabolites are excreted in the feces and urine [13]. Reportedly, it produces several types of metabolites in vivo, and they are more toxic than the active ingredient. A few examples of metabolites include IMI-NH (desnitroimidacloprid), resulting from imidacloprid, THI-NH (desyanothiacloprid) from thiacloprid, ACENH (desyanoacetamiprid) from acetamiprid, CLO-dm (desmethyl clothianidin), CLO-urea, and NG-F (methylguanidine) from clothianidin [13]. The α4β2 nAChR subtype is the main target of NPs; however, NPs have mild effects on other subtypes as well. In addition, some NP metabolites show an affinity for subtypes other than those targeted by the parent molecules [13]. The α4 and β2 subtypes of the mammalian nAChR are found not only in the nervous system, but also in a wide range of tissues, such as the ovary and testis [19,20,21].

In recent years, NPs have been reported to cause colony collapse disorder among honeybees (a phenomenon in which honeybees die or disappear in large numbers) [22,23,24]. Moreover, when administered to experimental animals, it crosses the intestinal mucosa, blood–brain barrier, and placenta, producing a wide range of symptoms that appear to be related to the central nervous system, autonomic ganglia, and neuromuscular junction [13]. The widespread environmental toxicity of NPs has thus attracted attention, and various studies have been conducted on this topic.

### 2.2. NPs in the Aquatic Environment

Aquatic organisms play an important role in maintaining aquatic ecosystems and are sensitive to changes in the environment. The types of organisms in aquatic ecosystems differ from region to region; they are also important food sources for fish and birds [25]. Karube et al. [26] reported that acetamiprid (maximum value 43.7 μg/L) detected in pond water at levels higher than the environmental standards may have led to reduced numbers of dragonfly larvae (*Sympetrum maculatum*) in the Tono region of Gifu prefecture, where populations of dragonflies were found until the 2000s. In addition, Hayasaka et al. [27] reported that residual insecticides leaked from the soil and exerted toxic effects on aquatic organisms. As a result, there was a decrease in the number of *Chironomus* larvae, which are the food source for dragonflies. Furthermore, it has been pointed out that the agricultural use of NPs in the past may have affected the yield of *Japanese smelt* by reducing the number of zooplankton [28]. There are concerns about the impact of NPs on ecosystems in the aquatic environment, and pesticides used in fields and orchards will likely have a significant impact on aquatic organisms in rivers. It has also been suggested that NPs affect not only the aquatic environment, but also terrestrial fauna, such as insects; the number of insects that are the food source for birds is reduced by NPs, and this may harm the bird population [29]. Thus, NPs may harm various ecosystems.

The toxicity of NPs toward aquatic insects is widely known. For *Cloeon dipterum*, the median lethal dose after chronic exposure was reported to be lower than that of the median lethal dose after 24 h of exposure [30]. In *Gammarus kischineffensis,* the median lethal dose of thiamethoxam after a 4-day exposure period was one-twentieth of that after acute exposure [31]. These studies indicated that NPs exert delayed chronic toxicity toward aquatic organisms [32] and that chronic exposure to low concentrations of NPs in the water may pose a problem for aquatic organisms. Therefore, it is important to investigate the concentrations of NPs in the aquatic environment.

### 2.3. NP Concentrations in River Water in Japan

Several studies have reported the concentrations of different NPs in Japanese rivers; data on the concentrations and collection month are summarized in Table 1. The items described in the queried papers were not consistent. Therefore, to compare data among seasons, NP species reports with complete information on river names, water sampling times, and those that investigated two or more NP species were targeted. In addition, as the time of use of NPs differs depending on the region, the month with the highest concentration of NPs was investigated (Table 1).

The rivers in Japan are managed by the Ministry of Land, Infrastructure, Transport, and Tourism in the case of first-class water systems, by prefectural governors in the case of second-class water systems, and by mayors in the case of independent water systems. Most rivers with data on the concentration of NPs are first-class water systems and are primarily rivers flowing through large cities (Table 1). In the Tsurumi [33], Sagami [34], Kaname [35,36], and Kuzuryu [37] river systems, imidacloprid, acetamiprid, dinotefuran, and clothianidin were detected at their maximum concentrations during the period from May to August (Table 1). These four NP species are the active ingredients in pesticides recommended by many agricultural cooperatives. Thiacloprid and nitenpyram were either not detected or were detected at maximum concentrations that were lower than those of the other species (Table 1). These two NP species have a comparatively lower estimated domestic distribution volume than the other species [40] and are not used extensively in Japan. Thiamethoxam is often used as an insecticide in fields and orchards other than paddy fields, but its time of use varies depending on the cultivar, and therefore, there may have been regional differences in the month of detection.

### 2.4. Safety of River Water in Japan

In Japan, pesticide registration standards have been established to prevent risks to animals and plants present in the living environment of water bodies (aquatic plant and animal standards) [41] and to prevent the pollution of river water that is used as drinking water (river water pollution standards) [42]. The two pesticide registration criteria have been set based on the results of assessments of the impact of the pesticides on animal/human health and the ecosystem conducted at the time of pesticide registration. In addition, the Food Safety Commission of the Cabinet Office evaluates health risks and sets the acceptable daily intake (ADI) [43] and acute reference dose (ARfD) values [43]. These reference values are summarized in Table 2.

The maximum detected concentrations of the NPs were below the aquatic plant and animal standards and river water pollution standards for all rivers (Table 1 and Table 2). In addition, a previous study focused on first-class water systems that flow through urban areas, and many surveys have been conducted with reference to drinking water. The Sagami, Tama [38], and Kuzuryu rivers are used as sources of drinking water; details regarding NP concentrations in these rivers are presented in Table 1. For example, if a person weighing 60 kg drinks 10 L of unpurified river water in one day [44], a total of approximately 2.7 μg of dinotefuran is ingested (the highest concentration of NP species detected in the Sagami, Tama, and Kuzuryu rivers was 0.27 μg/L). This value is much lower than the ADI (for a human weighing 60 kg; 13,200 μg) and ARfD (72,000 μg) values for dinotefuran and remains low when the maximum concentrations of dinotefuran in the Sagami, Tama, and Kuzuryu rivers are considered. Similarly, the daily ingestion values according to the highest concentrations of other NP species detected in the Sagami, Tama, and Kuzuryu rivers were also lower than their corresponding ADI and ARfD values. However, NPs have been reported to show delayed chronic toxicity in aquatic organisms [32]. The median lethal dose after chronic NP exposure in *Cloeon dipterum* was reported to be 0.30 μg/L for thiacloprid, 0.32 μg/L for imidacloprid, and 0.8 μg/L for thiamethoxam [30]. In a laboratory study on *Chironomus dilutus* larvae, the median lethal dose for 14 days was 1.52 μg/L for imidacloprid, 2.41 μg/L for clothianidin, and 23.60 μg/L for thiamethoxam; the 40-day median lethal doses of the same insecticides for *Chironomus dilutus* were 0.39, 0.28, and 4.13 μg/L, respectively [45]. Based on these data and the maximum concentrations of the NPs in each river (Table 1), it is clear that the NP levels in the Tsurumi and Kaname rivers are sufficiently high to adversely affect *Cloeon dipterum.* Detrimental effects on this organism may potentially translate to deleterious effects on the aquatic ecosystem. Thus, it is important to study the chronic toxicity of NPs in various aquatic organisms and to continue monitoring the concentrations of NPs in rivers.

### 2.5. NPs and Endocrine Disruptors

An endocrine disruptor (ED) is defined as “an exogenous substance or mixture that alters the function(s) of the endocrine system and consequently causes adverse health effects in an intact organism, its progeny, or (sub) populations of organisms” [46]. EDs are thought to be toxic primarily to the human reproductive system (genital cancer and decreased sperm count), immune system (asthma and atopic dermatitis), and nervous system (developmental disorders). It has also been reported that the sensitivity to EDs is particularly high in the fetal stage and during childhood [47]. Although only persistent pollutants, such as polychlorinated biphenyl (PCBs), dioxins, and DDT, were originally identified as EDs [48], many other chemicals from different groups, including non-persistent pesticides, phenols, and phthalates, have since been identified as EDs [49,50,51,52,53,54,55]. EDs, including pesticides, are currently being actively researched. In experiments with rodents, it has been reported that the administration of NPs adversely affects the nervous [14,15,56] and reproductive [15,56,57,58,59,60,61,62,63,64,65,66,67,68,69,70,71,72,73] systems. Moreover, the administration of imidacloprid to birds (*Amandava amandava*) adversely affects the hypothalamus–hypophysis–gonad axis [74,75]. Thus, NPs have direct and indirect adverse effects on the reproductive system, and their endocrine-disrupting effects are concerning [71,74,76,77].

### 2.6. Effects of NPs on the Testis

Mice and rats have been used as test animals to elucidate the toxic effects of imidacloprid, thiacloprid, clothianidin, and acetamiprid on the male reproductive system (Table 3). Many of these reports suggest testicular toxicity from NP administration [56,57,58,59,60,61,62,63,64,65,66,67,72,73]. The most commonly observed major testicular toxicities include decreased serum testosterone, decreased expression of testosterone metabolism-related genes, and decreased sperm count [56,57,58,59,60,61,62,63,64,65,66,67]. Testosterone is secreted by Leydig cells in the interstitium upon stimulation by the luteinizing hormone (LH), which is released from the anterior pituitary gland, and testosterone, activin, and the androgen-binding protein (secreted by Sertoli cells upon stimulation by the follicle-stimulating hormone [FSH]) also promote spermatogenesis. Therefore, testosterone plays an important role in maintaining a healthy reproductive system in men.

As spermatids appear after puberty in the testis, the environment in the testis changes significantly after attaining sexual maturation. Bal et al. [58,59] administered imidacloprid at 0.5, 2, and 8 mg/kg doses to rats and reported that in 1- and 8-week-old animals, the epididymal sperm concentration decreased at the 8 and 2 mg/kg doses, respectively, and the serum testosterone concentration decreased at the 0.5 and 8 mg/kg doses, respectively. Mohamed et al. [60] reported that NP toxicity-related disorders, such as decreased epididymal sperm and serum testosterone concentrations, are more pronounced in mature rats. Bal et al. [62] reported that when 1-week-old rats were administered with 2, 8, and 32 mg/kg of clothianidin, the highest dose led to decreased epididymal sperm and serum testosterone concentrations. Yanai et al. [66] reported that prenatal and early postnatal exposure to a no-observed-adverse-effect level (NOAEL) dose of clothianidin led to a reduction in the number of germ cells in juvenile male mice. However, 8-week-old rats exhibited no changes in epididymal sperm and serum testosterone concentrations upon being administered with 24 mg/kg of clothianidin [72]. Kong et al. [63] reported that when acetamiprid was administered to 5-week-old rats at 10 and 30 mg/kg doses, the epididymal sperm concentration decreased at the 10 mg/kg dose, whereas serum testosterone and plasma LH concentrations decreased and increased at the 30 mg/kg dose, respectively. Arican et al. [64] reported that when acetamiprid was administered to 8-week-old rats at 12.5, 25, and 35 mg/kg doses, the epididymal sperm concentration decreased at the 25 mg/kg dose, and the plasma LH and FSH concentrations increased at the 12.5 mg/kg dose of acetamiprid. Sensitivity to clothianidin and acetamiprid appears to be more pronounced in juvenile rats than in mature rats. Therefore, the results of the administration of NPs to animals may vary depending on the NP species and the age of the animals. However, the susceptibility results on imidacloprid and acetamiprid were inconsistent among different studies and need to be investigated further in the future.

The susceptibility to NP toxicity for mature and juvenile animals differs based not only on the type of NP, but also on the type of animal. Yuan et al. [64] reported that mice administered with imidacloprid showed a decreased expression of testosterone-related enzyme genes at a dose of 5 mg/kg and decreased serum testosterone concentrations and the induction of spermatogenic disorders at 30 mg/kg. In a rat imidacloprid administration experiment, 1 mg/kg led to decreased serum testosterone concentrations [60], and 2 mg/kg led to decreased epididymal sperm concentrations [58]. Terayama et al. [57] reported that an average dose of 2.6 mg/mouse of acetamiprid reduced the expression of testosterone-related enzymes and spermatogenesis-related genes in mice. In a rat acetamiprid administration experiment, it was reported that the epididymal sperm concentration decreased at a dose of 25 mg/kg [67]. Therefore, sensitivity to NPs was greater in rats than that in mice; thus, this susceptibility differed even among different rodent types.

Morphological observations showed that the administration of imidacloprid at a 45 mg/kg dose or more in rats led to decreased or no spermatogenesis, spermatocyte depletion, and the occurrence of mild interstitial edema of the testis [61,73]. In addition, when clothianidin was administered to mice and acetamiprid was administered to rats at 10 mg/kg or more, vacuolar degeneration of the seminiferous tubules was observed [56,63]. Maternal clothianidin exposure at approximately NOAEL in mice affected the number of germ cells in juveniles and induced their depletion in a highly susceptible individual mouse [66]. However, no changes were observed in the number of Sertoli cells, the tubule diameter, and the Leydig cell volume [66]. Thus, age at the time of exposure to NPs may affect the seminiferous epithelium. Edema of the interstitium and vacuolar degeneration in the seminiferous tubules in the testis are the morphological manifestations of NP toxicity, and these manifestations are precursors to developing spermatogenic disorders.

### 2.7. Effects of NPs on the Ovary

To elucidate the toxic effects of NPs on the female reproductive system, imidacloprid and clothianidin were administered to mice and rats; the results of various studies on this topic are summarized in Table 4. Reports suggest that NP administration leads to ovarian toxicity [68,69,70]. Mzid et al. [69] reported that in 10-week-old rats that received 50, 200, or 300 mg/kg of imidacloprid, the relative volume of atretic follicles decreased, whereas the relative volume of total follicles and the follicular diameter at different stages (primary, secondary, tertiary, and pre-ovulatory) increased. Moreover, the serum 17β-estradiol levels decreased at doses of 50 mg/kg or higher. Kapoor et al. [68] administered 5, 10, and 20 mg/kg of imidacloprid to 1-week-old rats and reported that there was an increase in serum FSH and a decrease in serum LH and progesterone in animals that received a 20 mg/kg dose. Imidacloprid damages follicles and exerts more toxicity in juvenile rats than in mature rats.

### 2.8. Mechanisms Underlying the Reproductive Toxicity of NPs

The toxic effects of NPs on the ovary and testis may result in follicular, testosterone secretion, and spermatogenesis disorders. Oxidative stress is one of the mechanisms underlying this toxicity. Oxidative stress is caused by an imbalance between active oxygen species and the antioxidant capacity; such stress is exerted when the production of reactive oxygen species (ROS) exceeds the antioxidant capacity of the living body or when there is a decrease in the cellular antioxidant capacity. ROS damage cell nucleic acids, proteins, and lipids, causing cell death and damage to various organs [78]. ROS can be categorized as a singlet oxygen, superoxide, hydrogen peroxide, and hydroxyl radicals. ROS are removed by antioxidant-related enzymes, such as superoxide dismutase (SOD), catalase (CAT), glutathione peroxidase (GPx), glutathione S-transferase (GST), and glutathione reductase (GR), and antioxidants, such as reduced glutathione (GSH), α-tocopherol (vitamin E), and ascorbic acid (vitamin C). It is thought that ROS damage cell nucleic acids, proteins, and lipids in the testis and ovary and cause damage by increasing oxidative stress.

Mitochondrial damage is one of the main causes of NP-induced ROS production [79]. NPs can cause DNA damage, apoptosis, protein oxidation, and lipid peroxidation in non-target organisms by altering mitochondrial Ca^2+^ homeostasis, inhibiting mitochondrial respiration, and inducing ROS production. The Ca^2+^ concentration plays an important role in maintaining mitochondrial membrane permeability, whereas neonicotinoids, including imidacloprid, and clothianidin, can increase intracellular Ca^2+^ levels [80,81], thereby inducing ROS production, which changes the mitochondrial membrane permeability.

The administration of imidacloprid led to increased levels of ROS, advanced oxidation protein products (AOPP; peroxidation protein marker), malondialdehyde (MDA; lipid peroxide marker), lipid peroxidation (LPO; lipid peroxide marker), 8-hydroxy-2′-deoxyguanosine mRNA (8-OhdG; DNA damage marker), and 8-oxoguanine glycosylase mRNA (OGG1; DNA repair marker) and decreased levels of SOD, CAT, GPx, GST, GR, and GSH in the testis (Table 3) [58,59,60,61,73]; imidacloprid administration also resulted in increased levels of AOPP, MDA, and LPO and decreased levels of SOD, CAT, GPx, GSH, and vitamin E in the ovary (Table 4) [68,69]. The administration of thiacloprid led to increased AOPP and MDA levels and reduced GSH, SOD, CAT, and GPx levels in the testis (Table 3) [65]. The administration of clothianidin lowered GSH in the testis (Table 3) [62] and also lowered GPx4 immunoreactivity in the ovaries (Table 4) [70]. The administration of acetamiprid led to increased MDA and TOS (total oxidant status) and decreased GST and TAS (total antioxidant status) in the testis (Table 3) [67]. The administration of imidacloprid, thiacloprid, clothianidin, and acetamiprid led to lowered levels of antioxidant enzymes and antioxidants and elevated levels of oxidative stress markers. Therefore, it is anticipated that similar phenomena underlie the toxic effects of other NPs. Imidacloprid administration led to BAX (B-cell lymphoma 2-associated X apoptotic protein) immunopositivity in testicular spermatogonial cells [60]; thiacloprid administration led to the appearance of a smear (a hallmark of necrosis) without ladder formation on agarose gel analysis (DNA fragmentation test) for cells in the testis [65]. Clothianidin administration increased the apoptotic index in the germinal epithelium of the testis and led to the fragmentation of seminal DNA [62]; acetamiprid administration increased the apoptotic index (detected by the terminal deoxynucleotidyl transferase dUTP nick-end labeling (TUNEL) method) in testicular tissue [66]. Therefore, it is accepted that the administration of NPs increases oxidative stress and causes cell death in reproductive tissues.

## 3. Conclusions

In Japan, although NPs reach the rivers, their levels in aquatic bodies are below the environmental standard. However, chronic toxicity of aquatic insects and reproductive toxicity of rodents have been reported with NP exposure, and there are many species differences in susceptibility to NP toxicity. Further research on the effect of NP exposure on various organisms is therefore necessary. One of the causes of NP toxicity is oxidative stress. In addition, globally, there is a clear trend towards the reduced use of NPs. The Japanese aquatic plant and animal standards were revised in 2006 to increase the variety of evaluation experiments. *Daphnia magna* was used in the experiment at the time of registration of the pesticide; however, the standard value was very high because the species has an extremely low sensitivity to NPs among the planktons [82]. Therefore, the results of experiments on *Chironomid*, *Paratya improvisa*, and *Gammaridea* have also been evaluated; the values for dinotefuran, nitenpyram, imidacloprid, thiacloprid, fipronil (2017), and acetamiprid (2018) were revised to lower values [41,42]. In addition, the Japanese water pollution standards for fipronil were revised in 2018. However, to increase the intended insecticidal effects, the Japanese food residue standard values for clothianidin and acetamiprid were relaxed in 2015 [83]. The relaxation of food residue standards is contrary to the global trend. It is necessary to continue monitoring the water system concerning NP levels in the future.

## Figures and Tables

**Table 1:** Maximum concentrations and sampling months of neonicotinoid pesticides in Japanese rivers, as reported in previous studies.

Region	Sampling Point	Time of Water Sampling	Compound	Maximum Concentration (mg/L)	Sampling Month with Maximum Concentration	Reference
Kanagawa	Upstream–downstream of the Tsurumi River	May–December 2009	Imidacloprid	0.42	July	[33]
			Acetamiprid	0.06	June	
	Midstream–downstream of the Sagami River	Late April 2014–Mid-March 2015	Imidacloprid	0.104	June	[34]
			Acetamiprid	0.023	- ^1^	
			Thiacloprid	0.002	-	
			Thiamethoxam	0.202	-	
			Dinotefuran	0.048	-	
			Clothianidin	0.085	June	
	Upstream–downstream of the Kaname River	April–December 2017	Imidacloprid	0.836	June	[35]
			Acetamiprid	0.779	July	
			Thiacloprid	0.006	November	
			Thiamethoxam	0.029	November	
			Nitenpyram	N.D. ^2^	-	
			Dinotefuran	0.373	August	
			Clothianidin	0.482	May	
	Midstream–downstream of the Kaname River *	April–August, November 2018, March 2019	Imidacloprid	0.095	June	[36]
			Acetamiprid	0.004	June	
			Thiacloprid	Unclear	-	
			Thiamethoxam	0.011	May	
			Nitenpyram	Unclear	-	
			Dinotefuran	0.043	August	
			Clothianidin	0.053	June	
			Fipronil	0.037	June	
Fukui	Downstream of the Kuzuryu River	April–November 2018	Imidacloprid	0.055	May	[37]
			Acetamiprid	0.0012	August	
			Thiacloprid	0.0012	-	
			Thiamethoxam	0.076	May	
			Nitenpyram	N.D.	-	
			Dinotefuran	0.27	August	
			Clothianidin	0.13	August	
			Fipronil	0.0045	-	
Tokyo	Midstream of the Tama River	April–May 2017	Imidacloprid	0.0084	-	[38]
			Acetamiprid	0.00094	-	
			Thiacloprid	0.00045	-	
			Thiamethoxam	0.0037	-	
			Nitenpyram	N.D.	-	
			Dinotefuran	0.0089	-	
			Clothianidin	0.047	-	
			Fipronil	0.0018	-	

Papers cited in Table 1 were selected from the Google Scholar website (https://scholar.google.co.jp/, accessed on 28 July 2022). * These values were read from the line graph published in the article. ^1^ -: no data were published in the cited study. ^2^ N.D.: pesticide was not detected. Table 1 is a revised version of a table from a previous study [39].

**Table 2:** Environmental standards and food standards for various neonicotinoid pesticides in Japan (as of 29 June 2021).

Standard Type	Standard Name	Imidacloprid	Acetamiprid	Thiacloprid	Thiamethoxam	Nitenpyram	Dinotefuran	Clothianidin	Fipronil	Reference
Environmental standard (μg/L)	Registration withholding standards for agricultural chemicals with reference to prevention of toxicity to aquatic plants and animals	1.9	2.5	3.6	3.5	11	12	2.8	0.024	[43]
	Registration withholding standards for agricultural chemicals with reference to prevention of water pollution	150	180	31	47	1400	580	250	0.5	
Food standard (μg/kg)	Acceptable daily intake (ADI)	57	71	12	18	530	220	97	0.19	
	Acute reference dose (ARfD)	100	100	31	500	600	1200	600	20	

Table 2 is a revised version of a table from a previous study [39].

**Table 3:** Various effects of neonicotinoid pesticides determined in previous reports on testes.

Subject	Target Animal	Age at Initial Exposure	Dose (Per Day)	Exposure Period	Administration Method	Conclusion						Reference
						Testis Weight	Spermatogenesis	Androgen	Oxidative Stress	Cell Death	Other	
Imidacloprid	Wistar albino rat	8–9 weeks	0.5, 2, 8 mg/kg	90 days	Oral (gavage)	N.S. ^1^	•Decreased epididymal sperm concentration (2, 8 mg/kg) and sperm motility (8 mg/kg) •Increased apoptosis indexes in the germinal epithelium of the testis, fragmentation of seminal DNA, and abnormal sperm rate (head) (8 mg/kg).	•Decreased serum testosterone (8 mg/kg)	•Decreased GSH in the testis (8 mg/kg)	-	•Decreased relative weights of epididymis, right cauda epididymis, and seminal vesicles (8 mg/kg) •Increased levels of oleic acid, linoleic acid (2, 8 mg/kg), stearic acid, and arachidonic acid in the testis (8 mg/kg)	[58]
	Wistar albino rat	7 days	0.5, 2, 8 mg/kg	90 days	Oral (gavage)	N.S.	•Decreased epididymal sperm concentration (8 mg/kg) •Increased proportion of abnormal sperm (head, tail, and total values) (8 mg/kg).	•Decreased serum testosterone (0.5, 2, 8 mg/kg)	•Increased MDA and decreased GSH in the testis (8 mg/kg)	-	•Increased testicular levels of palmitic, palmitoleic, stearic, oleic, linoleic, dihomo-γ-linolenic, arachidonic, docosapentaenoic acids as well as total lipid values (0.5, 2, 8 mg/kg).	[59]
	Wistar albino rat	8 weeks	45, 90 mg/kg	28 days	Oral (gavage)	N.S.	•Decreased total epididymal sperm count, sperm motility, and live sperm count (45, 90 mg/kg) •Increased proportion of sperm with abnormal head or tail morphology (45, 90 mg/kg) •Increased proportion of abnormal sperm (45, 90 mg/kg) •Increased γ-GT activity, LDH-x, SDH in sperm suspension (45, 90 mg/kg)	•Decreased testicular 3β-HSD, 17β-HSD, and testosterone and serum testosterone (45, 90 mg/kg)	•Decreased protein concentration, reduced GSH, SOD, and GPx activity (45, 90 mg/kg) and CAT and GST activity in the testis (90 mg/kg) •Increased LPO and percent ROS-positive cells in a dose-dependent manner in the testis (45, 90 mg/kg)	-	•Decreased weight of epididymis (45, 90 mg/kg)	[61]
	Sprague–Dawley rat	4 weeks and 7 weeks	1 mg/kg	65 days	Oral (- ^3^)	Decreased(4 and 7 weeks)	•Decreased epididymal sperm concentration, motility, intact seminal DNA percentage, and viable sperm percentage (4 and 7 weeks).	•Decreased serum testosterone, serum LH, serum estradiol, 3β-HSD mRNA, and NR5A1 mRNA (4 and 7 weeks).	•Increased 8-OHdG and OGG1 mRNA (4 and 7 weeks)	•Intense BAX-positive immunolabeling of the spermatogonial cell cytoplasm with moderate signals in the remaining spermatozoa (7 weeks)•Intense BAX-positive immunolabeling of the spermatogonial cell cytoplasm (3 weeks)	-	[60]
	Wistar rat	12–14 weeks	16.9 mg/kg	28 days	Oral (-)	N.D. ^2^	-	-	•Decreased GST, GR, GPx, CAT, and SOD activity and mean TTH in the testis •Increased testicular AOPP and MDA levels	-	-	[73]
	ICR mouse	6 weeks	3, 10, 30 mg/L	70 days	Oral (-)	Decreased(30 mg/L)	• Decreased seminiferous tubule score determined by the Johnson scoring method in the testis (30 mg/L)	•Decreased serum testosterone, estradiol, and aromatase; testicular TC (30 mg/L) and serum LDL-C (10, 30 mg/L)•Decreased SR-B1 (3 mg/L), StAR, PBR, AR (10, 30 mg/L), P450scc, 3β-HSD (3, 10, 30 mg/L), P45017a, 17β-HSD, and HMG-CoA (30 mg/L) mRNA	-	-	•Imidacloprid showed binding affinity to the androgen receptor	[64]
Thiacloprid	Wistar albino rat	Adult	22.5 mg/kg	30 days	Oral (gavage)	N.S.	•Decreased epididymal sperm concentration, sperm motility, sperm viability, and testicular DNA contents •Increased proportion of abnormal sperm	-	•Decreased SOD, CAT, and GPx activity and SOD, CAT, and GPx mRNA in the testis•Increased GSH, MDA, and AOPP in the testis	•Smear (a hallmark of necrosis) without ladder formation on agarose gel by the DNA fragmentation method in the testis	-	[65]
Clothianidin	Wistar albino rat	7 days	2, 8, 32 mg/kg	90 days	Oral (gavage)	N.S.	•Decreased epididymal sperm concentration (32 mg/kg) •Increased proportion of sperm with abnormal head or tail morphology (8 and 32 mg/kg) •Increased proportion of abnormal sperm (8 and 32 mg/kg)	•Decreased serum testosterone protein (32 mg/kg)	•Decreased GSH (32 mg/kg)	•Increased apoptotic index in the germinal epithelium of the testis and fragmentation of the seminal DNA (32 mg/kg)	•Increased palmitic acid (8 mg/kg), arachidonic acid, docosapentaenoic acid (8, 32 mg/kg), testicular tissue cholesterol level (32 mg/kg), palmitoleic acid, and total lipid values (2, 8, 32 mg/kg) in the testis	[62]
	Wistar albino rat	8–9 weeks	2, 8, 24 mg/kg	90 days	Oral (gavage)	N.S.	N.S.	N.S.	-	-	•Decreased relative weights of epididymis (2, 8, 24 mg/kg), right cauda epididymis (8 mg/kg), and seminal vesicles (2, 8 mg/kg) •Increased LPO (measured as TBARS level), palmitic acid, linoleic acid, arachidonic acid, and cholesterol levels in the testis (2, 8, 24 mg/kg)	[72]
	C57BL/6NCrSlc Mouse	8 weeks	10, 50, 250 mg/kg	28 days	Oral (gel intake)	N.S.	•Abnormal GPx4 immunoreactivity in Sertoli cells of the seminiferous tubules that also showed marked degeneration (250 mg/kg)	-	-	-	-	[56]
	C57BL/6NCrSlc Mouse(Male offspring postnatally on day 14)	Gestational day 1	10, 50 mg/kg	Approximately 30 days	Oral (gel intake)	N.S.	•Decreased number of germ cells per seminiferous tubule (50 mg/kg)	N.S.	-	-	-	[66]
Acetamiprid	Sprague–Dawley rat	5 weeks	10, 30 mg/kg	35 days	Oral (gavage)	Decreased(10, 30 mg/kg)	•Decreased number of spermatids and epididymal sperm (10, 30 mg/kg)	•Decreased plasma testosterone (30 mg/kg), Leydig cell number, StAR mRNA, Cyp11a1 mRNA, and 3β-HSD mRNA (10, 30 mg/kg) in the testis, cAMP (30 mg/kg), and ATP in the Leydig cells (10, 30 mg/kg)•Increased plasma LH (30 mg/kg), MDA, and NO in the Leydig cells (10, 30 mg/kg)	-	-	-	[63]
	A/J mouse	3 weeks	Average 2.6 and 21.4 mg/mouse	180 days	Oral (through water intake)	N.S.	•Decreased Ki-67 (21.4 mg) and Top2a (2.6 and 21.4 mg) mRNA	•Decreased LH receptor, StAR, Cyp11a1, HSD17b3 (21.4 mg), and Cyp17a1mRNA (2.6 and 21.4 mg) in the testis	-	-	•Acetamiprid concentrations detected: 63.9 pg/mL in the serum and 7.1 pg/mL in the testis (21.4 mg) •Decreased nAChRα4 (21.4 mg) and nAChRα7 mRNA (2.6 and 21.4 mg)	[57]
	Sprague–Dawley rat	8–10 weeks	12.5, 25, 35 mg/kg	90 days	Oral (gavage)	N.S.	•Decreased epididymal sperm concentration (25, 35 mg/kg) and seminiferous tubule score determined by the modified Johnson scoring method and proliferation index determined by the PCNA method in the testicular tissue (12.5, 25, 35 mg/kg). •Increased inhibin B (25 mg/kg) in the plasma	•Decreased cholesterol (25, 35 mg/kg) in the plasma •Increased FSH (12.5, 25, 35 mg/kg) and LH (12.5, 25 mg/kg) in the plasma	•Decreased GSH and TAS in the plasma and GSH and TAS in the testis (12.5, 25, 35 mg/kg) •Increased MDA (12.5, 25, 35 mg/kg) and TOS (35 mg/kg) in the plasma and P8MDA (25, 35 mg/kg) and TOS (35 mg/kg) in the testis	• Increased apoptotic index determined by the TUNEL method in the testicular tissue (12.5, 25, 35 mg/kg)	-	[67]

Table 3 is a revised version of a table presented in a previous study [57]. Papers cited in Table 3 were selected from the PUBMED website (https://pubmed.ncbi.nlm.nih.gov, accessed on 28 July 2022). ^1^ N.S.: difference not significant. ^2^ N.D.: testis weight not detected. ^3^ -: no data were published in the cited study. AOPP, advanced oxidation protein product; ATP, adenosine triphosphate; AR, androgen receptor; BAX, BCL2-associated X protein; cAMP, cyclic adenosine monophosphate; CAT, catalase; Cyp11a1, cytochrome P450 family 11 subfamily A member 1; Cyp17a1, cytochrome P450 family 17 subfamily A; FSH, follicle-stimulating hormone; GPx, glutathione peroxidase; GPx4, glutathione peroxidase 4; GR, glutathione reductase; GSH, glutathione; GST, glutathione S-transferase; HMG-CoA, 3-hydroxy-3-methyl-glutaryl CoA synthase; HSD17b3, 17β-hydroxysteroid dehydrogenase 3; LDH-x, lactate dehydrogenase-x; LDL-C, low-density lipoprotein cholesterol; LH, luteinizing hormone; LPO, lipid peroxidation; MDA, malondialdehyde; NO, nitric oxide; NR5A1, nuclear receptor family 5 group A member 1; nAChRα4, nicotinic acetylcholine receptor α4; nAChRα7, nicotinic acetylcholine receptor α7; OGG1, 8-oxoguanine glycosylase; PBR, peripheral benzodiazepine receptor; PCNA, proliferating cell nuclear antigen; P450scc, cytochrome P450 cholesterol side-chain cleavage enzyme; P45017a, cytochrome P450 family 17 subfamily A; ROS, reactive oxygen species; SDH, sorbitol dehydrogenase; SOD, superoxide dismutase; SR-B1, scavenger receptor class B member 1; StAR, steroidogenic acute regulatory protein; TAS, total antioxidant status; TC, total cholesterol; Top2a, DNA topoisomerase II alpha; TOS, total oxidant status; TTH, total thiol level; γ-GT, γ-glutamyltranspeptidase; 3β-HSD, 3β-hydroxysteroid dehydrogenase; 8-OHdG, 8-hydroxy-2′-deoxyguanosine; 17β-HSD, 17β-hydroxysteroid dehydrogenase.

**Table 4:** Effects of neonicotinoid pesticides on the ovary reported in previous studies.

Subject	Target Animal	Age at Initial Exposure	Dose (Per Day)	Exposure Period	Administration Method	Conclusion						Reference
						Ovary Weight	Oogenesis	Female Hormones	Oxidative Stress	Cell Death	Other	
Imidacloprid	*Rattus norvigicus* Wistar strain	7 days	5, 10, 20 mg/kg	90 days	oral (gavage)	Decreased (20 mg/kg)	- ^1^	•Increased serum FSH (20 mg/kg) •Decreased serum LH and progesterone (20 mg/kg)	•Increased LPO in the ovary (20 mg/kg) •Decreased GSH content, SOD, CAT, and GPX activity in the ovary (20 mg/kg)	-	-	[68]
	Rat	10–12 weeks	50, 200, 300 mg/kg	60 days	oral (gavage)	Decreased (50, 200, 300 mg/kg)	•Increased relative volume of atretic follicles against total follicles (50, 200, 300 mg/kg) •Increased follicular diameters at the primary (200, 300 mg/kg), secondary (200, 300 mg/kg), tertiary (50, 200, 300 mg/kg), and pre-ovulatory (50, 200, 300 mg/kg) stages	•Decreased serum 17β-estradiol (50, 200, 300 mg/kg)	•Increased ovarian AOPP and MDA (50, 200, 300 mg/kg) •Decreased vitamin E, GSH, SOD, CAT, and GPX activity in the ovary (50, 200, 300 mg/kg)	-	-	[69]
Clothianidin	C57BL/6NCrSlc Mouse (postnatal female offspring at 3 weeks and 10 weeks)	Gestational period day 1.5	65 mg/kg	The dams (pregnant mouse) were given soft gel with or without CLO from gestational day 1.5 to postnatal day 21 period.	oral (gel intake)	Decreased (3 weeks)	N.S. ^2^	•Decreased 17-hydroxyprogesterone (17-OH progesterone) and corticosterone in the blood (10 weeks) •Decreased activation of genes in the steroid hormone biosynthesis pathway in the ovary (3 weeks)	•Decreased GPx4 immunoreactivity in the ovary (3 weeks and 10 weeks)	-	-	[70]

Papers cited in Table 4 were selected from the PUBMED website (https://pubmed.ncbi.nlm.nih.gov, accessed on 28 July 2022). ^1^ -: no data were published in the cited study. ^2^ N.S.: no significant difference. AOPP, advanced oxidation protein product; CAT, catalase; FSH, follicle-stimulating hormone; GPx, glutathione peroxidase; GPx4, glutathione peroxidase 4; GSH, glutathione; LH, luteinizing hormone; LPO, lipid peroxidation; MDA, malondialdehyde; SOD, superoxide dismutase.

## Data Availability

Not applicable.

## Abbreviations

NPs	neonicotinoid pesticides
DDT	dichlorodiphenyltrichloroethane
nAchR	nicotinic acetylcholine receptor
CYP	cytochrome P
ADI	acceptable daily intake
ARfD	acute reference dose
ED	endocrine disruptor
PCBs	polychlorinated biphenyl
LH	luteinizing hormone
NOAEL	no-observed-adverse-effect level
AOPP	advanced oxidation protein product
ATP	adenosine triphosphate
AR	androgen receptor
BAX	BCL2-associated X protein
cAMP	cyclic adenosine monophosphate
CAT	catalase
Cyp11a1	cytochrome P450 family 11 subfamily A member 1
Cyp17a1	cytochrome P450 family 17 subfamily A
FSH	follicle-stimulating hormone
GPx	glutathione peroxidase
GPx4	glutathione peroxidase 4
GR	glutathione reductase
GSH	glutathione
GST	glutathione S-transferase
HMG-CoA	3-hydroxy-3-methyl-glutaryl CoA synthase
HSD17b3	17β-hydroxysteroid dehydrogenase 3
LDH-x	lactate dehydrogenase-x
LDL-C	low-density lipoprotein cholesterol
LPO	lipid peroxidation
MDA	malondialdehyde
NO	nitric oxide
NR5A1	nuclear receptor family 5 group A member 1
nAChRα4	nicotinic acetylcholine receptor α4
nAChRα7	nicotinic acetylcholine receptor α7
OGG1	8-oxoguanine glycosylase
PBR	peripheral benzodiazepine receptor
PCNA	proliferating cell nuclear antigen
P450scc	cytochrome P450 cholesterol side-chain cleavage enzyme
P45017a	cytochrome P450 family 17 subfamily A
ROS	reactive oxygen species
SDH	sorbitol dehydrogenase
SOD	superoxide dismutase
SR-B1	scavenger receptor class B member 1
StAR	steroidogenic acute regulatory protein
TAS	total antioxidant status
TC	total cholesterol
Top2a	DNA topoisomerase II alpha
TOS	total oxidant status
TTH	total thiol level
γ-GT	γ-glutamyltranspeptidase
3β-HSD	3β-hydroxysteroid dehydrogenase
8-OHdG	8-hydroxy-2′-deoxyguanosine
17β-HSD	17β-hydroxysteroid dehydrogenase
AOPP	advanced oxidation protein product
vitamin E	α-tocopherol
vitamin C	ascorbic acid
